# ICORG 10-14: NEOadjuvant trial in Adenocarcinoma of the oEsophagus and oesophagoGastric junction International Study (Neo-AEGIS)

**DOI:** 10.1186/s12885-017-3386-2

**Published:** 2017-06-03

**Authors:** JV Reynolds, SR Preston, B O’Neill, L Baeksgaard, SM Griffin, C Mariette, S Cuffe, M Cunningham, T Crosby, I Parker, K Hofland, G Hanna, LB Svendsen, CL Donohoe, C Muldoon, D O’Toole, C Johnson, N Ravi, G Jones, AK Corkhill, M Illsley, J Mellor, K Lee, M Dib, V Marchesin, M Cunnane, K Scott, P Lawner, S Warren, S O’Reilly, G O’Dowd, G Leonard, B Hennessy, R Mc Dermott

**Affiliations:** 1 St. James’s Hospital and Trinity College Dublin, Dublin, Ireland; 2 Beaumont Hospital, Dublin, Ireland; 3 Royal Surrey County Hospital, Guildford, UK; 4 Velindre Cancer Centre, Cardiff, Wales UK; 5 Southampton Clinical Trials Unit, University of Southampton, Southampton, UK; 6 Rigshospitalet, Copenhagen, Denmark; 7 University Hospital C. Huriez Place de Verdun, Lille, France; 8 Royal Victoria Infirmary, Newcastle, UK; 9 St Mary’s Hospital and Imperial College London, London, UK; 10 Irish Clinical Oncology Research Group (ICORG), Dublin, Ireland

**Keywords:** Oesophageal, Oesophagogastric junction, Adenocarcinoma, Neoadjuvant chemoradiation therapy, Neoadjuvant chemotherapy

## Abstract

**Background:**

Neoadjuvant therapy is increasingly the standard of care in the management of locally advanced adenocarcinoma of the oesophagus and junction (AEG). In randomised controlled trials (RCTs), the MAGIC regimen of pre- and postoperative chemotherapy, and the CROSS regimen of preoperative chemotherapy combined with radiation, were superior to surgery only in RCTs that included AEG but were not powered on this cohort. No completed RCT has directly compared neoadjuvant or perioperative chemotherapy and neoadjuvant chemoradiation. The Neo-AEGIS trial, uniquely powered on AEG, and including comprehensive modern staging, compares both these regimens.

**Methods:**

This open label, multicentre, phase III RCT randomises patients (cT2-3, N0-3, M0) in a 1:1 fashion to receive CROSS protocol (Carboplatin and Paclitaxel with concurrent radiotherapy, 41.4Gy/23Fr, over 5 weeks). The power calculation is a 10% difference in favour of CROSS, powered at 80%, two-sided alpha level of 0.05, requiring 540 patients to be evaluable, 594 to be recruited if a 10% dropout is included (297 in each group). The primary endpoint is overall survival, with a minimum 3-year follow up. Secondary endpoints include: disease free survival, recurrence rates, clinical and pathological response rates, toxicities of induction regimens, post-operative pathology and tumour regression grade, operative in-hospital complications, and health-related quality of life. The trial also affords opportunities for establishing a bio-resource of pre-treatment and resected tumour, and translational research.

**Discussion:**

This RCT directly compares two established treatment regimens, and addresses whether radiation therapy positively impacts on overall survival compared with a standard perioperative chemotherapy regimen Sponsor: Irish Clinical Research Group (ICORG).

**Trial registration:**

NCT01726452. Protocol 10-14. Date of registration 06/11/2012.

**Electronic supplementary material:**

The online version of this article (doi:10.1186/s12885-017-3386-2) contains supplementary material, which is available to authorized users.

## Background

Adenocarcinoma of the oesophagus and the oesophagastric junction (AEG) have markedly increased in incidence in the West over the last 30 years [1–3]. AEG tumours have been defined topographically by Siewert et al., with true oesophageal (AEG I) tumours arising in a background of reflux-induced specialised intestinal metaplasia, AEG II representing true cardia tumours, and AEG III denoting gastric tumours within 5 cm below the cardia and involving the junction [4]. The disease is often advanced at presentation, and the overall 5-year survival is very poor at between 10–20% [1]. For patients presenting with localised disease, several advances in standards of care have emerged in recent years which have improved disease management and survival, with an approximate 5-year survival of 35 to 50% for patients who can be treated with curative intent. First, comprehensive staging with computed tomography-^18^FDG positron emission tomography (PET-CT) and endoscopic ultrasound (EUS) has improved selection of patients for curative treatment [1, 5]. Second, the increasing centralisation of care within high-volume hospitals has increased focus on all aspects of quality assurance, with associated improvements in oncological and operative outcomes [6]. Finally, via a number of randomised controlled trials (RCTs), neoadjuvant and adjuvant therapies have been established as superior to surgery alone, and are increasingly the standard of care for locally advanced disease [7].

There are four key RCTs evaluating neo-adjuvant or perioperative chemotherapy compared with surgery alone. The RTOG 8911/Intergroup 0113 RCT randomised 440 patients, 54% with adenocarcinoma, to pre- and postoperative 5-Fluorouracil (FU) and cisplatin (Cis), and compared this regimen with surgery alone. No improvement in survival was evident [8, 9]. A similarly powered study of 802 patients conducted by the Medical Research Council in the UK, the OEO2 Trial, where 66% of patients had adenocarcinoma, randomised patients to two cycles of pre-operative Cis and FU, or surgery alone, and reported a significantly improved survival at 5 years [23% compared with 17% (*p* = .03)] [10, 11]. The Medical Research Council Adjuvant Gastric Infusional Chemotherapy (MAGIC) trial of 503 patients, although powered for gastric adenocarcinoma, included 11% with junctional and 14% with lower oesophageal adenocarcinoma, and compared 3 cycles of epirubicin, Cis and FU (ECF) before and after surgery with surgery alone [12]. The 5-year survival rate was 36% for combined modality therapy compared with 23% for patients with surgery alone (*p* = 0.009). The French ACCORD-07 provided similar results, recruiting 224 of a planned 250 patients, with 64% having junctional adenocarcinoma, and 11% with lower oesophageal adenocarcinoma [13]. Cis and FU was administered before and after (in responders) surgery, and the 5 year survival was 38% for combination therapy compared with 24% for surgery alone (*p* = 0.02). Accordingly, the MAGIC and ACCORD trials provide a significant level of evidence for perioperative chemotherapy in adenocarcinoma of the lower oesophagus and junction compared with surgery alone. Moreover, a new dimension was provided through the United Kingdom National Cancer Research Institute REAL2 trial [14]. This multicentre trial of patients with advanced inoperable or recurrent disease, using a 2×2 randomisation, showed the non-inferiority of substituting oral capecitabine (X) for infused FU, and oxaliplatin (O) for Cis, in the ECF regimen, with oxaliplatin associated with lower incidences of grade 3 or 4 neutropenia, renal toxicity and thromboembolic side effects.

Up to the recent publication of the CROSS trial, the interpretation of trials of combination chemotherapy and radiation therapy prior to surgery, and meta-analysis, was more difficult compared with trials using chemotherapy alone, for several reasons, including small underpowered studies, a mix of pathological types, variation in dose and fractionation across studies, inadequate pre-treatment staging, and poor outcomes in surgical only cohorts. In the CROSS trial (Chemoradiotherapy for Esophageal Cancer followed by Surgery Study), 366 patients, 75% with adenocarcinoma, were randomised to multimodal therapy (paclitaxel, carboplatin and 41.4 Gy/23 fractions) or surgery alone [15]. The median overall survival (OS) was 49 months in the multimodality arm compared with 24 months for surgery alone (*p* = 0.003), with a corresponding 5-year OS rate of 47% in the multimodality arm compared with 34% in the surgery cohort. The neoadjuvant regimen was completed by 162 (95%) of 171 patients, with a low recurrence of grade 3 or adverse effects [29 (17%) of 171 patients). Side-effects were few, 13 (8%) had grade 3 or worse haematological toxicity, and 18 (11%) had grade 3 or worse non-haematological toxicity. The R0 resection rate, reflecting complete surgical resection and negative margins, was 92% in the multimodality arm compared with 69% in the surgery only arm. Longer follow up showed reduced locoregional recurrences in the treatment arm, and to a lesser extent reduced systemic recurrences [16, 17]. At this time, accordingly, the CROSS trial provides strong support for the evidence-base supporting preoperative chemoradiation in oesophageal cancer, which, notwithstanding its widespread use, was somewhat inconsistent from earlier RCTs [18–21].

Studies comparing pre-or perioperative chemotherapy with preoperative chemoradiation are lacking. The Preoperative Chemotherapy or Radiochemotherapy in Esophagogastric Adenocarcinoma Trial (POET) is the only phase III RCT to address this question, where 119 patients with EUS staged (uT3-4, Nx, Mo) adenocarcinoma (AEG 1and II) received either preoperative induction chemotherapy (Cis, FU and leucovorin) or induction chemotherapy followed by chemoradiation (Cis and etoposide with 30Gy in 15 fractions of radiation therapy [RT]), and then surgery [22]. The trial was closed prematurely due to slow accrual, with 3 year survival in the multimodal arm of 47.4% compared with 27.7% in the chemotherapy arm (*p* =0.07.) However, the pathological complete response rate was significantly (15.6% vs 2% *p* = 0.03) improved with the addition of radiation, and consistent with other trials comparing multimodal therapy with surgery alone, the pathological node negative rate was significantly decreased (64.4% vs 37.7%; *p* =0.01).

The excellent results reported for the CROSS trial define this currently as the multimodal standard of care for oesophageal cancer, and the modified MAGIC regimen, chosen for comparison in Neo-AEGIS, is in common use in the UK and Europe for junctional adenocarcinoma. The Neo-AEGIS trial accordingly will compare regimens which have provided Level I evidence of their superiority compared with surgery alone, albeit not in trials focused exclusively on adenocarcinoma of the oesophagus and junction. The proposed trial will be unique in also including AEG type III in addition to AEG types I and II, this being enabled by the recent AJCC/UICC 7th edition staging of all junctional tumours as oesophageal, and harmonising nodal staging (N1-3) across what was formerly different nodal staging for oesophageal (AEG I and II) and gastric (AEG III)-derived adenocarcinoma [23]. The trial will also be the first to include CT-PET as the uniform standard baseline staging modality, as well as embedded quality assurance for delivery of RT.

## Methods and Design

### Study design (Fig. 1)

**Fig. 1:**
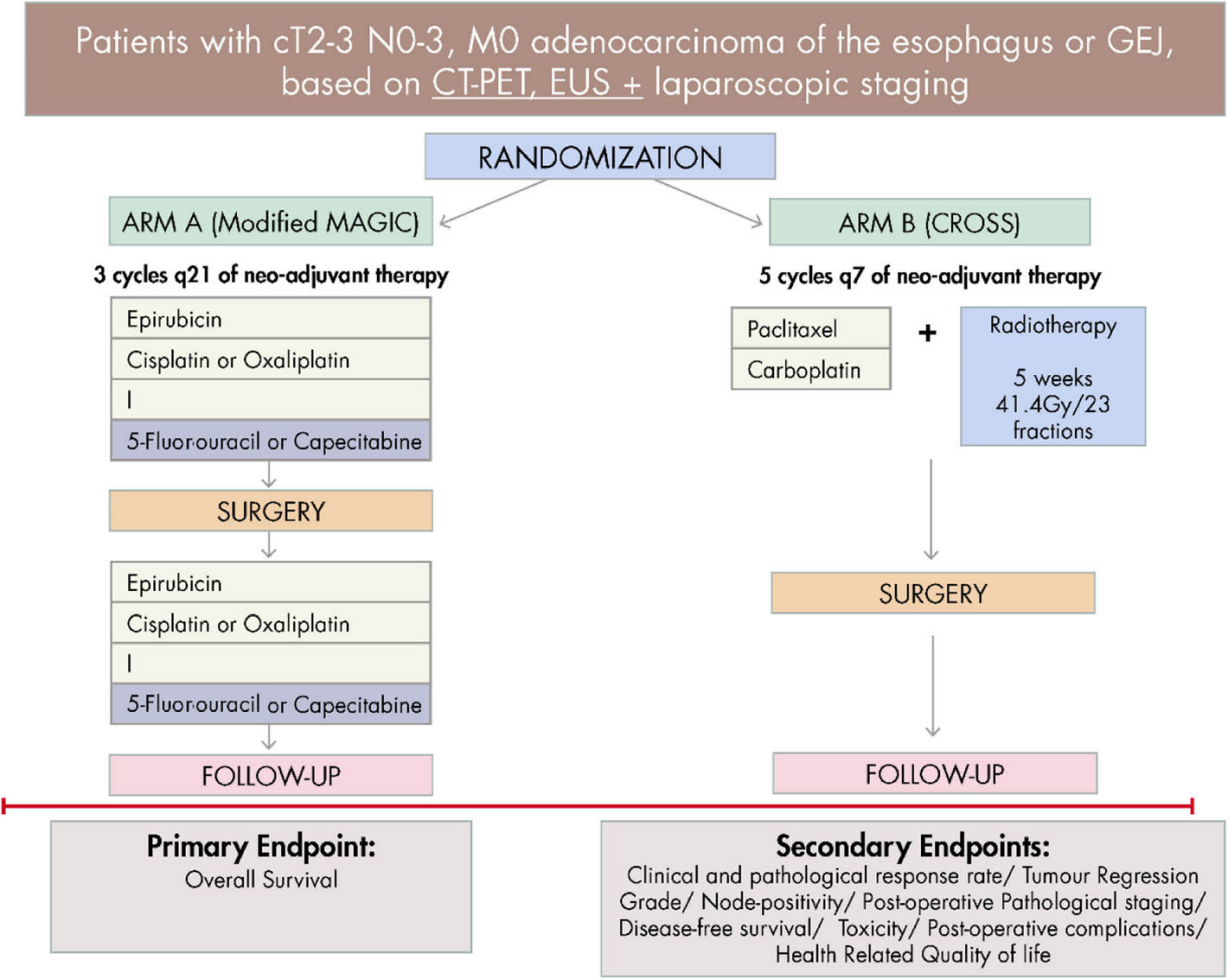
Schedule of enrolment, interventions, and assessments

Neo-AEGIS is a multicentre phase III open-labelled, randomised controlled trial. Eligible patients will be randomised in a 1:1 fashion between the modified MAGIC regimen (ECF/ECX or EOF/EOX) or the multimodality therapy (CROSS protocol), the latter with a modernised design and delivery of radiation therapy. The aim of this study is to compare the two Level-1 evidence based regimens exclusively in patients defined as high-risk for relapse. High-volume (i.e. > 30 resections per year) centres or nationally designated oesophageal cancer surgery centres will participate.

### Study objectives

The primary objective is to evaluate overall survival, calculated from the date of randomisation and an event registered on the date of death from any cause. Secondary objectives include the effect of both neoadjuvant regimens on clinical response rate (relief of dysphagia, improvement in health related quality of life, endoscopic regression, radiological response), tumour regression grade, surgical resection rate, pathological R0 resection, post-operative pathology including nodal involvement, disease-free survival, time to treatment failure, site of treatment failure, and toxicity. Toxicity of treatment will be graded according to the National Cancer Institute Common Terminology Criteria for Adverse Events (NCI-CTCAE) version 4.0 [24]. All post-operative complications will be captured for up to 90 days after surgery and graded according to the Clavien-Dindo classification system, and the Esophageal Complications Consensus Group (ECCC) consensus on standardisation of data collection for complications associated with oesophagectomy [25].

### Patient selection

Patients with histologically proven adenocarcinoma of the oesophagus or oesophago-gastric junction will undergo preoperative staging with endoscopy, and CT-PET. EUS will be performed in all cases except where near-complete dysphagia precludes assessment, and laparoscopy is recommended for locally advanced intra-abdominal disease. Patients, with pre-treatment clinical stage cT2-3, N0-3, M0, and tumours less than 8 cm in length, will be assessed for suitability for inclusion to this trial. Requirements include an ECOG performance status 0-2; an adequate absolute neutrophil count (ANC) >1.5x10^9^/l; white blood cell count > 3x10^9^/l; platelets > 100x10^9^/l; haemoglobin (Hb) > 9 g/dl (can be post-transfusion); a glomerular filtration rate > 60 ml/min calculated using the Cockcroft-Gault Formula; and serum bilirubin <1.5x Upper Limit of Normal (ULN); AST <2.5x ULN and ALP <3x ULN (ULN as per institutional standard); and adequate respiratory function, including an FEV1 > 1.5 L. For all patients an ejection fraction of greater than 50% is required, assessed by either echocardiogram or MUGA scan. Patients with a significant cardiac history (e.g. known ischemic disease, cardiomyopathy) must have in addition cardiac clearance by a cardiologist. In addition to exclusions based on not meeting above criteria, prior abdominal or thoracic radiotherapy, chemotherapy for gastrointestinal cancer, peripheral neuropathy, known HIV or other malignancies within 5 years are also exclusion factors.

## Treatment regimens

### Arm A – Modified Magic Chemotherapy Regimen

The regimen consists of 3 pre- and 3 postoperative cycles of RCF/ECX or EOF/EOX. Each cycle of chemotherapy lasts 21 days/3 weeks. The drugs used in the modified MAGIC regimen include Epirubicin intravenously (IV) (50 mg/m^2^; day 1), Cisplatin IV (60 mg/m^2^ with concurrent 500 ml 10% mannitol IV, day 1) or Oxaliplatin IV (130 mg/m^2^ day 1) and 5-Fluorouracil IV (200 mg/m^2^/d × 3 weeks)/or Capecitabine 625 mg/m^2^ twice daily for 3 weeks orally. The choice between administering Cisplatin or Oxaliplatin and 5 Fluorouracil or Capecitabine is at the discretion of the investigator.

Arm A: Modified MAGIC Regimen

**Table:** 

Drug	Dose	Administration	Days Given
Epirubicin	50 mg/m^2^	Slow IV push into the side arm of NaCl 0.9% drip	Day 1
Cisplatin with hydration - mannitol 10%^*^	60 mg/m^2^	Dilute in NaCl 0.9%.	
	500 mls	Administer over 2 h.	
OR		Infuse concurrently with cisplatin over 2 h	Day 1
Oxaliplatin	130 mg/m^2^	Dilute in Dextrose 5%. Administer over 2 h	
5-Fluorouracil	200 mg/m^2^/d	Given at a dose of 200 mg/m^2^/day by continuous infusion every day for 21 days/3 weeks	Daily for 21 days/3 weeks
OR			
Capecitabine	625 mg/m^2^ twice daily	Taken orally twice daily for 21 days	Daily for 21 days/3 weeks

### Arm B – CROSS protocol, Multimodal arm: Chemotherapy and Radiotherapy Regimen

Arm B consists of the multimodal arm, which includes a combination of chemotherapy and radiotherapy prior to surgery. The patient will receive 4 and a half weeks of radiation therapy (41.4 Gy/23 fractions/1.8 Gy per fraction) and 5 weekly cycles of chemotherapy. RT is administered on days 1-5, days 8-12, days 15-19, days 22-26 and days 29-31 inclusive. The patient will receive 5 cycles of chemotherapy, Paclitaxel 50 mg/m2 and Carboplatin Area Under Curve (AUC) =2 (calculated using the Calvert formula [26]), given by IV infusion on days 1,8,15,22, and 29.

Arm B: Chemotherapy

**Table:** 

Drug	Dose	Administration Details	Days Given
Dexamethasone	10 mg	Give IV half an hour before commencing Paclitaxel	Days 1, 8, 15, 22 and 29
Chlorphenamine	10 mg	Give IV half an hour before commencing Paclitaxel	Days 1, 8, 15, 22 and 29
Ranitidine	50 mg	Give IV half an hour before commencing paclitaxel	Days 1, 8, 15, 22 and 29
Paclitaxel	50 mg/m^2^	The total calculated dose of paclitaxel, infused over 1 h IV (PVC free).	Days 1, 8, 15, 22 and 29
Ondansetron	8 mg	Administer orally half an hour before carboplatin	Days 1, 8, 15, 22 and 29
Carboplatin Area Under Curve =2	As per Calculation	The total calculated dose of carboplatin, infused over 1 h IV.	Days 1, 8, 15, 22 and 29

## Radiotherapy

A planning CT scan will be performed following randomisation with intravenous contrast injection (providing adequate renal function). Patients will fast for 2 h and then drink 200mls of liquid 30 min prior to CT planning and prior to each treatment in an attempt to reproduce the same anatomical position of the stomach throughout treatment. Patients will be scanned and treated, in the supine position with arms above their head, knee support, and immobilisation with thermoplastic device or vacuum cushion as per local protocols. The scan will fully include target and all organs at risk (lungs, liver, kidneys and stomach).

Diagnostic information for the purpose of defining the target volume will be taken from the diagnostic CT scan, EUS (referenced to CT identifiable structure) and PET/CT. Target volume definition (TVD) will be divided into (i) middle third - defined here as primary tumour epicentre between 24 cm and 32 cm *ab* oral, and (ii) lower third and oesophagogastric junction (Siewert types AEG I-III) - defined here as primary tumour epicentre from 32 cm *ab* oral to sub-cardia, which is expected represent the vast majority of cases in this trial. Where the passage of the EUS scope across the tumour has not been possible, findings from diagnostic endoscopy and PET imaging will be used to define the disease centre.

TVD is described in detail in the protocol along with a contouring atlas. In brief: The gross tumour volume (GTV) consists of the gross tumour (**GTV**_**T**_) and regional malignant lymph nodes (**GTV**_**N**_). GTV_**T**_ **and GTV**_**N**_ include the entire circumference of the oesophagus, and are combined without a margin to form **GTV**_**TN**_ using the Treatment Planning System (TPS). The clinical target volume (CTV) is made up of the GTV including a margin for occult disease. There are three CTVs in this protocol, **CTV**_**A**_, **CTV**_**B**_ and **CTV**_**COMB**._ **CTV**_**A**_ is defined by an isotropic margin of 0.5 cm around **GTV**_**TN**_. **CTV**_**B**_ will comprise of the ‘fat pad’ around the oesophagus. The ‘fat pad’ is contoured at the same levels as the **GTV**_**TN**_, for 3 cm superior and inferior to **GTV**_**T**_. Thus, **CTV**_**B**_ encompasses the entire GTV with a cranio-caudal margin to account for occult submucosal spread of tumour. The ‘fat-pad’ is encompassed to cover subclinical nodal disease. The Planning Target Volume (**PTV**) is created using the TPS via the expansion of **CTV**_**COMB**_ by an isotropic margin of 10 mm (5 mm IM and 5 mm SM), in all dimensions.

Below the junction **GTV**_**T**_ does not include the entire circumference of the stomach at the level of disease, but just the gross tumour. Where **GTV**_**N**_ includes nodes in the abdomen, **CTV**_**B**_ is extended to include that entire nodal station. No other abdominal nodal stations will be electively included. Spinal Cord, Spinal Cord PRV (planning organ at risk volume) Lungs Heart, Liver and both Kidneys are delineated as Organs at risk (OARs). Dose-volume histograms of these structures will be obtained for all patients. Where possible, the volume of both lungs that receive 20Gy or more will not exceed 25% of combined lung volume (V20Gy lung < 25%). The volume of the heart that receives 40Gy will not exceed 30% of the heart volume (V40Gy heart < 30%) and volume of heart that receives 25Gy will not exceed 50% of the heart volume (V25Gy heart < 50%) (these are optimal objectives – to be achieved where possible but at lower priority than other objectives). The volume of the liver that receives 30Gy will not exceed 30% of the total liver volume (V30Gy liver < 30%). The maximum dose to the spinal cord may not exceed 45Gy (Dmax (0.03 cc) < = 45Gy). The maximum dose to 1 cm^3^ of the spinal cord PRV may not exceed 40Gy (D1cm^3^ < 40Gy). If the PTV lies close to or overlaps with the Spinal Cord PRV, the treating clinician may discretionally allow a point maximum dose up to 45Gy. Alternatively, they may report a PTV compromise.

A single-phase coplanar 3D-conformal treatment plan will be calculated on the planning CT scan, tailored to achieve optimal PTV coverage while respecting the dose volume constraints. The plan will typically be delivered from 3–5 gantry angles (though the number of gantry angles is not mandated) and utilize only megavoltage photon energies. The treatment fields will conform to the target via the use of multileaf collimators. Megavoltage photon energies ≥ 6MV is mandatory. The use of cone beam CT matched to planning CT scans is mandatory within this study. 4D planning will be encouraged within the Neo-AEGIS trial but is not mandated.

A meticulous Radiotherapy Quality Assurance (RTQA) programme has been developed for the Neo-AEGIS trial. Pre-trial RTQA Procedures are as follows: All centres who wish to participate in Neo-AEGIS must satisfactorily complete a pre-accrual outlining benchmark case of a mid and lower oesophageal cancer case. Outlines will be compared against a consensus reference volume. Each centre must submit a satisfactory plan for a pre-outlined patient. A Radiotherapy Process Document must be produced describing technical details of all trial patient processes for that centre. For on-trial RTQA, radiotherapy should commence within 15 days of signing consent, meaning that prospective/real-time review is not practical. Instead, there will be timely-retrospective review of the outline and plan for the first patient case from each centre (the RTQA team will give feedback to the centre within 2 weeks of the start of treatment). A second case will also be reviewed should there have been an issue with the first case. A random allocation of 10% of all outlines and plans will be submitted from all centres for timely-retrospective review as above. This will be the responsibility of each national Radiotherapy Principal Investigator (PI) [Brian O’Neill (Irl); Tom Crosby (UK), Kenneth Hofland (Dk)].

### Adverse Events (AE)

Adverse events will be recorded on the Adverse Event case report form and must be graded using the National Cancer Institute's Common Terminology Criteria for Adverse Events (NCI CTCAE) version 4.0 [24]. All non-serious and serious AE occurring in each patient will be reported up to 30 days after the last dose of study drug has been received. The last dose of study drug is defined as the last dose of study drug the patient receives during the last post-surgery chemotherapy cycle (ARM A) and the last dose of chemotherapy or radiation therapy the patient receives prior to the scheduled surgery (ARM B). Postoperative complications up to 90 days will be reported separately.

### Surgery

The operation will be performed between 3 and 10 weeks following completion of neoadjuvant therapy. Radical en-bloc resection around the tumour as well as a regional lymphadenecomy is the operative goal, however, approaches may vary depending on tumour location and institutional preference and may include en-bloc transthoracic, both 2 and 3 stage, with 2-field lymphadenectomy, minimally invasive oesophagectomy or hybrid approaches, transhiatal approaches, and an extended total gastrectomy and abdominal lymphadenectomy for AEG type III junctional tumours. All post-operative complications will be recorded on the case report form for up to 90 days after surgery. The highest severity complication will be graded according to the Clavien-Dindo classification system and the recent International Consensus on Standardization of Data Collection for Complications, agreed by the Esophageal Complications Consensus Group [25, 26].

### Pathology

Pathological assessment is performed as per standard guidelines and is based on the UICC/TNM 7th edition (2010) [23]. In patients treated with neo-adjuvant therapy, the extent of residual carcinoma in the oesophagectomy specimen will be assigned to one of five categories as per Mandard et al [27]. TRG1 represents a complete response (pCR); TRG2 represents rare residual cancer cells scattered throughout the fibrosis; TRG3 represents an increase in the number of residual cancer cells, but fibrosis still predominate, TRG4 represents residual cancer cells outgrowing fibrosis and TRG5 represents a complete absence of regression change. The lymph node dissection should contain at minimum 15 nodes. The circumferential resection margins (R status) will be determined by both the residual tumour classification system outlined by the Royal College of Pathology (RCP) (R1 denotes tumour < 1 mm) from margin, and the College of American Pathologists (CAP) classification where R1 denotes actual margin involvement [28, 29].

### Follow-up

An initial post-operative visit will occur within 2 weeks -1 month post-discharge from hospital post- surgery, or sooner as patients’ condition determines. After the initial post -operative visit to the surgical team, the patient in Arm A will be assessed for adjuvant chemotherapy and will be referred back to the treatment oncologist for completion of treatment. Follow up visits with the trial centre will occur at 3 months, 6 months, 9 months and 12 months for the 1st year and 6 monthly thereafter or until progression of disease is documented or death, whichever occurs earlier. CT or CT-PET scan (as per institutional standard practice) will be carried out 1 year post operatively or earlier if deemed clinically necessary as per surgeon and annually thereafter. Recurrence of disease should be documented by appropriate imaging and biopsies where appropriate.

### Statistical analysis

The study primary end point is overall survival. The study design is with a two-sided alpha level of 0.05, and an estimated 80% power to detect -year increase in overall survival of 10% i.e. 43% to 53%, through the use of the CROSS protocol, and with initial evaluation at 3 years of follow up of the last patient. A two-sided log rank test with an overall sample size of 594 subjects (of which 297 are in group 1 and 297 are in group 2) achieves 80% power at a 0.05 significance level to detect a difference of 0.10 between 0.43 and 0.53-the proportions surviving in groups 1 and 2, respectively. This corresponds to a hazard ratio of 1.55. The data will be analysed three years after the last patient enters the study. The proportion of non-evaluable patients is assumed to be 10%. These results are based on the assumption that the hazard rates are proportional. Recruitment will occur over 5 years.

Disease-free survival will be calculated from randomization to the first event (i.e. local recurrence or progression, distant recurrence, or death from any cause), and overall survival will be calculated from randomization to death. Kaplan-Meier curves for disease-free and overall survival will be compared with the use of the log-rank test on an intention to treat basis. Hazard ratios will be calculated with the use of a Cox regression model including treatment alone (primary analysis) and after adjustment for prognostic factors of interest. Categorical data will be compared with the use of chi-squared tests, with a test for trend over ordered categories (T, N, etc). Tumour measurements will be compared with the use of appropriate parametric t-tests or nonparametric Mann-Whitney tests. All tests will be two sided. The primary analysis will be performed on an intention to treat (ITT) basis, that is, for all patients randomised into the study, regardless of whether they received treatment or what treatment they received. A per-protocol analysis, excluding patients who did not sufficiently comply with the protocol (in terms of exposure to treatment, availability of measurements and major protocol violations) will supplement the ITT analysis as a secondary analysis.

Interim futility analyses will be based on the rule of Freidlin et al with a Linear 20% Inefficacy Boundary (LIB20) [30]. This rule provides the opportunity to terminate early for evidence that the experimental arm will not prove superior, but protects against aggressive early termination for treatment effect sizes smaller than planned. To allow for the situation in which the experimental treatment is nontrivially less efficacious than the control arm, there will be an early ‘harm’ look based on 25% of events (deaths), followed by two further interims based on 40% and 70% of information. The results of the futility analyses will be reported to the DSMB.

### Ethical and regulatory considerations

The trial has been submitted and approved by a multi-centre research ethics committee in each of the participating countries, and received clinical trials authorisations from regulatory authorities. This trial was approved by the joint research ethics committee of Tallaght Hospital/St James’ Hospital (REC reference: 2016-02 List 5 [11]). The study will be conducted in accordance with ethical principles founded in the Declaration of Helsinki [31]. In each centre, the lead investigator will be responsible for identification, recruitment, data collection, completion of case report forms, follow-up of study patients and adherence to study protocol. Informed consent will be obtained from participants by the local lead investigator using a standardised consent form and participant information sheet (Additional file 1: Appendix A). A trial steering committee (TSC) is responsible for the day-to-day running of the trial (composition: Additional file 2: Appendix B), and an independent data and safety monitoring board (DSMB) with no competing interests has been appointed to review the trial approximately every 6 months. An interim analysis is planned after recruitment of approximately 200 patients, at the discretion of the DSMB. The Chief Investigator (JVR) is responsible for the design and conduct of Neo-AEGIS, and in combination with the project team in ICORG is responsible for the preparation of protocol and revisions, preparation of investigators literature and case report forms, and organization of steering committee meetings. ICORG is responsible for study planning, data master file, data verification and randomization. Randomisation will be generated via a computer-generated random numbers sequence without stratification. Upon completion of the enrolment form, the allocation will be generated via contacting ICORG. The TSC are responsible for agreement of the final protocol and any revisions. Revisions are communicated to all lead investigators via the TSC. All lead investigators are members of the TSC and one lead investigator per country is nominated as national coordinator. Authorship will be defined as per International Committee of Medical Journal Editors guidelines [32]. Results will be communicated at relevant international conferences, via publication and on the clinical trial registry. Data are collected using the individual trial case number on standard case report forms collated centrally by ICORG and personal information will not be individually identifiable. The final trial dataset will be available to study investigators but will not be analysed per centre.

### Withdrawal criteria

Treatment delay beyond 3 weeks is acceptable for treatment related toxicity, in which case a delay of up to 6 weeks is permitted before treatment is considered intolerable and the patient is withdrawn. Other situations where a study treatment may need to be interrupted for other clinical reasons will be considered. However, if an interruption of study treatment of more than 3 weeks is anticipated, the study chief investigator will be notified in advance, and further advice sought with regards to the appropriateness of restarting study treatment. Other criteria for which treatment will be withdrawn include: disease progression before completion of study treatment; withdrawal of consent for treatment and/or study participation; unacceptable treatment-related toxicity or adverse events as judged by either the patient’s treating physician or the Chief Investigator; pregnancy; and patient non-compliance.

## Discussion

The most recent updated meta-analysis of RCTs for oesophageal cancer, published in 2011, and including the CROSS trial, concludes that “a clear advantage of neoadjuvant chemoradiotherapy over neoadjuvant therapy has not been established” [7]. Consequently, there is no standard of care internationally, and established practice in many countries often mirrors the outcomes of RCTs conducted, with neoadjuvant chemotherapy practiced in the UK and France, whereas a multimodality approach is more standard in the Netherlands, Australia, North America, Ireland and Hong Kong [33]. The reports of the CROSS trial have clearly established benchmark outcomes and accepted toxicities that will result in broad application internationally [15, 16]. Notwithstanding, the effects of the CROSS regimen for adenocarcinoma are markedly inferior to its impact on squamous cell cancer. This is highlighted in a recent report on long term outcomes of CROSS, with a median follow up of 84 months in surviving patients, where the Hazards Ratio [HR 95% Confidence intervals (CI)] was 0.48 (0.28–0.83; *p* = 0.008) for squamous cell cancer and 0.73 (0.55–0.98; *p* = 0.038) for adenocarcinoma [16]. Although significant, the margin of benefit for adenocarcinoma compared with surgery does not establish a standard of care based on the trial evidence, and lends weight to the need to establish clear evidence whether the addition of radiation to the multimodal regimen is superior to systemic chemotherapy in the context of locally advanced but resectable disease.

Both CROSS and the MAGIC trials were large studies and adequately powered, neither however was powered solely on adenocarcinoma of the oesophagus and junction. In the CROSS trial the outcomes in the surgery only group were superior to any previously reported outcomes from RCTs, highlighting evolving major improvements in the overall quality of oesophageal cancer treatment, including staging, risk assessment, and all aspects of treatment including operative outcomes. The Neo-AEGIS trial is powered with a 10% difference chosen based on approximate estimates relating to available trial data for adenocarcinoma alone, and an adjustment in addition for the fact that MAGIC and CROSS were not contemporaneous trials. All measures of quality assurance that underpin outcomes in the modern high-volume oesophageal centre will be applied in Neo-AEGIS, including state of the art staging, quality assurance in radiation therapy and pathology, and surgery only performed by high volume surgeons. The trial is also unique in including all junctional adenocarcinoma, including AEG III, which were excluded from CROSS, but are all grouped together with the most recent TNM staging and denoted as “oesophageal” tumours. The trial also uses a new international consensus on defining oesophageal operative complications [26]. An added value of the trial, not expounded in this publication, is also the opportunity to collect tissue and blood samples for storage in the bio bank for future international collaborative translational research, in particular targeted towards response prediction and prognosis.

## Additional files

Additional file 1 Appendix A: Description of data: copy of consent form (DOCX 60 kb)

Additional file 2 Appendix B: - Description of data: Membership of Trials Steering Committee (DOCX 22 kb)

## Abbreviations

3D: Three dimensional
AE: Adverse Event
AEG: Adenocarcinoma of the oesophagus and junction
ALT: Alanine aminotransferase
ANC: Absolute Neutrophil Count
AST: Aspartate aminotransferase
AUC: Area under curve
CAP: College of American Pathologists
CI: Confidence interval
Cis: Cisplatin
CROSS: Chemoradiotherapy for Esophageal Cancer followed by Surgery Study
CT-PET: Computerised Tomography-Positron Emission Tomography
CTV: Clinical Target Volume
CTV_A_: Isotropic margin of 0.5 cm around GTV_TN_
CTV_B_: Fat pad 3 cm superior and inferior to GTVT
Dk: Denmark
Dmax: Maximum Dose
DSMB: Drug and safety monitoring board
E: Epirubicin
ECCG: Esophageal Complications Consensus Group
ECOG: Eastern Cooperative Oncology Group
EUS: Endoscopic ultrasound
FEV_1_: Forced Expiratory Volume in 1 second
FU: 5-Fluorouracil
GTV: Gross Tumour Volume
GTV_N_: Gross Tumour Volume of Regional lymph nodes
GTV_T_: Gross Tumour Volume of tumour
Gy: Gray
Hb: Haemoglobin
HIV: Human Immunodeficiency Virus
HR: Hazards Ratio
ICORG: Irish Clinical Oncology Research Group
IDMC: Independent Data Monitoring Committee
Irl: Ireland
ITT: Intention to treat
IV: Intravenous
LIB20: Linear 20% Inefficacy Boundary
M: Metre
MAGIC: Medical Research Council Adjuvant Gastric Infusional Chemotherapy
Mg: Milligram
Ml: Millilitre
MUGA: Multigated Acquisition Scan
MV: Megavoltage
NaCl: Sodium Chloride
NCI-CTCAE: National Cancer Institute Common Terminology Criteria for Adverse Events
Neo-AEGIS: NEOadjuvant trial in Adenocarcinoma of the oEsophagus and oesophagoGastric junction International Study
O: Oxaliplatin
OAR: Organ at risk
OS: Overall Survival
pCR: Complete pathological response
PI: Principal investigator
POET: Preoperative Chemotherapy or Radiochemotherapy in Esophagogastric Carcinoma Trial
PRV: Planning organ at risk volume
PTV: Planning Target Volume
R1: Microscopic positive resection margin
RCP: Royal College of Pathology
RCT: Randomised Controlled Trial
REC: Research Ethics Committee
RT: Radiation therapy
RTOG: Radiation Therapy Oncology Group
RTQA: Radiotherapy quality assurance
TMG: Trial Management Group
TNM: Tumour/Nodes/Metastases
TPS: Treatment planning system
TRG: Tumour Regression Grade
TSC: Trial Steering Committee
TVD: Target Volume Definition
UICC: Union for International Cancer Control
UK: United Kingdom
ULN: Upper Limit of Normal
X: Capecitabine
