# Supplementary material for: ICORG 10-14: NEOadjuvant trial in Adenocarcinoma of the oEsophagus and oesophagoGastric junction International Study (Neo-AEGIS)
Source: BMC Cancer. 2017 Jun 3;17:401. doi: 10.1186/s12885-017-3386-2 (PMC5457631; doi:10.1186/s12885-017-3386-2)
Supplement: Supplementary file 1 — Appendix A: Description of data: copy of consent form (DOCX 60 kb) [file 12885_2017_3386_MOESM1_ESM.docx]

**Appendix A: Copy of consent form**

For publication purposes, the consent does not contain the original header and footer.

Header states: To be printed on hospital headed paper

Footer contains page number and also includes: ICORG 10-14 –Neo-AGIS Study

Sample Patient Information Leaflet and Consent Form ICORG Version 6 27-Aug-2015

Hospital: [Name] Version x dd-mmm-yyyy

**Sample Patient Information Leaflet and Consent Form**

**ICORG 10-14 Patient Information Leaflet and Consent Form**

**Study Title:** Neo-AEGIS (NEOadjuvant trial in Adenocarcinoma of the oEsophagus and oesophagoGastric junction International Study): Randomised Clinical Trial of neoadjuvant and adjuvant chemotherapy (Modified MAGIC regimen) vs. neoadjuvant chemoradiation (CROSS protocol) in adenocarcinoma of the oesophagus and oesophago-gastric junction

**Study Number/ICORG Number:** 10-14

**Study Doctor Name:** <Investigator Name>

**Study Doctor Address:** <Hospital Site Address>

**Sponsor Name and Address:** The Irish Clinical Oncology Research Group (ICORG), 60 Fitzwilliam Square, Dublin 2, Ireland.

**Introduction**

You are being invited to take part in a Clinical Research Study. In order to decide whether or not you should agree to be part of this study, it is important for you to understand why the study is being done, what it will involve, as well as the possible risks, benefits and discomforts. This process is known as Informed Consent.

This Patient Information Leaflet gives detailed information about the Clinical Research Study that your study doctor will discuss with you. Please take time to read the following information carefully and make sure you fully understand it. If you would like to know more about anything mentioned in this leaflet, or have any questions about this research study, please be sure to ask your study doctor or nurse.

Thank you for reading this. Please take your time to decide whether or not you wish to take part.

**Who is organising the research?**

This study is organised and sponsored by the Irish Clinical Oncology Research Group (ICORG).

**What is the purpose of the study?**

Your study doctor has explained to you that you have been diagnosed with cancer of the oesophagus or at the junction between the oesophagus and stomach.

Surgery, radiotherapy and chemotherapy are all used on their own or in combination to treat cancer of the oesophagus, or the junction between the oesophagus and the stomach. It has become more common in recent years to give either chemotherapy alone or combined with radiation therapy before surgery for these cancers. We do not know which is better, and perhaps there is no difference.

The aim of this clinical research study is to compare and evaluate two well-established standard of care treatments for cancer of the oesophagus in order to establish if one treatment is better than the other or not. These treatments are tried and tested approaches, but they have never been compared directly, and that is the purpose of this study. Only a well-designed study such as this will answer that question and hopefully define the better approach for the future. The two treatments are:

1. Chemotherapy before and after surgery (treatment A)
2. Chemotherapy and radiotherapy before surgery (treatment B)

**What will happen during the study?**

Before treatment:

Before your treatment begins you will undergo the standard investigations that are performed on any patient who presents with cancer of the oesophagus or its junction with the stomach. These tests will help the study doctor to diagnose and stage your cancer. You may have to be admitted into hospital for these tests.

- You will have a complete physical examination and your medical history will be taken.
- You will have an **endoscopy** (also referred to as oesophago-gastro-duodenoscopy (OGD)) test done where a tube called an endoscope is passed down into your oesophagus (food pipe). The study doctor can see inside your oesophagus and take a small sample of tissue (biopsy).
- You will also have a **PET-CT** scan done. PET stands for Positron Emission Tomography and CT (also known as a CAT scan) stands for Computerized Tomography. This scan is a special type of x-ray that builds up a detailed picture of the inside of your body. A small amount of a mildly radioactive substance is injected into a vein and you will wait about an hour before the scan is taken. As any abnormal areas absorb more of the radioactive substance than normal areas these will show up on the scan. This body scan also shows up whether there has been any spread of the cancer.
- You will also have an **endoscopic ultrasound** (EUS) done. This is similar to the endoscopy but in this case there is an ultrasound probe connected to the end of the endoscope and passed along your oesophagus. This allows deeper views of your oesophagus and the surrounding area to be looked at.
- You may also have a **laparoscopy** done. This is a procedure, which is done under general anaesthetic to allow your study doctor to look inside your abdomen to assess your tumour.
- You will also have standard blood tests taken and you may have tests done to check your lungs.
- You will also have a MUGA scan or echocardiogram test done to check your heart.
- The nurse may give you a questionnaire to complete before treatment to assess your current quality of life.

These tests are done to make sure that you can take part in this study and that it will be safe for you to be treated on this study. Even if you decide not to take part in this study, most of these tests are necessary to diagnose and treat your illness.

Study Consent and Randomisation:

Once the above tests and investigations have been completed and if you decide to participate in the study you will start treatment. If you decide not to participate in the study, this is not a problem; your study doctor will continue to treat you with the standard treatment for your illness.

If you consent to participate, you will be enrolled on the study and assigned randomly to one of two treatment groups. You may be assigned to receive either treatment A; chemotherapy before and after your surgery, or treatment B: chemotherapy and radiotherapy before your surgery. These treatments will be explained in more detail later in this leaflet.

You or your study doctor have no influence over which treatment you will be allocated to receive, this is all done through a computerised system based at ICORG. You have a 50:50 chance of being in either group.

During treatment:

During your treatment you will be assessed by the oncology and/or radiation oncology study doctors and nurses. A complete physical examination and your medical history will be taken. Your height and weight will also be measured. You will also have some blood tests taken to check your blood counts. It is important that you let the study doctors and nurses know how you are feeling whilst on treatment and if you are experiencing any side effects. The nurse will also ask you to complete a questionnaire after your chemotherapy (and radiotherapy if you have been assigned to treatment B) to assess your current quality of life.

Before surgery:

Once initial treatment is completed you will undergo a CT or PET-CT scan and OGD to see how your tumour has responded to treatment. Once these are complete you will return to the out-patients department for review by your study doctor to assess your suitability to progress to surgery. If this is appropriate you will be given a provisional date for surgery and will await admission into hospital. You will be fully prepared and educated regarding what to expect before your surgery.

After surgery:

Once your surgery is complete and you are discharged from hospital, you will return to the out-patient department after approximately one month for a check-up.

If you are on treatment A you will be re-assessed at this point for some more chemotherapy treatment after your surgery. If you are well enough to re-start chemotherapy you will be given an appointment to go back to see your oncology study doctor again. In addition, you will return for check-ups every 3 months for a year, and then every 6 months thereafter.

If you are on treatment B you will not receive chemotherapy after your surgery. After your check-up one month after your surgery you will continue to return for check-ups every 3 months for a year, and then every 6 months thereafter.

At these check-up visits you will undergo a routine examination by the study doctor and the nurse will give you a quality of life questionnaire to complete. Blood tests will only be done as necessary. You will undergo a CT scan and OGD approximately one year after your surgery or sooner if deemed necessary by your study doctor.

**How many people will take part in the study?**

About 594 patients from centres in four European countries; Ireland, the UK, Denmark and France, will take part in the study. Of these about 200 patients are expected to come from centres in Ireland.

**How long will I be on the study?**

You will be in the study for the duration of the treatment; Treatment A: 6-7 months, Treatment B: 3-4 months., You will be followed-up by the study team at the hospital for a minimum of 3 years after your surgery by contacting you every six-months thereafter.

**Do I have to take part?**

It is up to you to decide whether or not to take part. If you do decide to take part you will be asked to sign the attached consent form and you will be given a copy of this information leaflet to keep. If you decide to take part but later change your mind, you are free to withdraw at any time without giving a reason. This will not affect the standard of care you receive. Likewise, your study doctor may decide to withdraw you from the study if it is in your best interest.

**What are the alternatives for treatment?**

If you decide not to take part in this study, you will receive the best treatment currently available. Your study doctor will discuss with you other treatment options available to patients with your type of cancer and explain the risks and benefits of these options to you.

Your other choices may include:

- Getting the standard of care treatment for your cancer without being in this study
- Taking part in another research study
- Getting no treatment

**What are the possible side effects of participating in this study?**

There are no added side-effects from participating in the study over and above the standard side-effects of these treatments which will be discussed in detail with you by your oncology and radiation oncology study doctors and nurses.

**What will my treatment involve if I am allocated to receive treatment A?**

If you have been assigned to treatment A, you will receive 6 cycles of chemotherapy; 3 cycles **before** your proposed surgery and 3 cycles **after** your surgery. Chemotherapy| is the use of anti-cancer (cytotoxic) drugs to destroy cancer cells. You will be given an information booklet on chemotherapy from your study nurse specialist; this booklet is given as part of standard care.

**What drugs are used in the chemotherapy regimens of Treatment A?**

Chemotherapy drugs that are commonly used in combination (known as a regimen) to treat cancer of the oesophagus or its junction with the stomach are as follows|. The regimen names ECF/ECX and EOF/EOX come from the initials of the chemotherapy drugs used.

Your study doctor will decide which regimen is suitable for you.

| **ECF** | **ECX** | **EOF** | **EOX** |
| --- | --- | --- | --- |
| [**E**pirubicin](http://www.macmillan.org.uk/Cancerinformation/Cancertreatment/Treatmenttypes/Chemotherapy/Individualdrugs/Epirubicin.aspx) | [**E**pirubicin](http://www.macmillan.org.uk/Cancerinformation/Cancertreatment/Treatmenttypes/Chemotherapy/Individualdrugs/Epirubicin.aspx) | [**E**pirubicin](http://www.macmillan.org.uk/Cancerinformation/Cancertreatment/Treatmenttypes/Chemotherapy/Individualdrugs/Epirubicin.aspx) | [**E**pirubicin](http://www.macmillan.org.uk/Cancerinformation/Cancertreatment/Treatmenttypes/Chemotherapy/Individualdrugs/Epirubicin.aspx) |
| [**C**isplatin](http://www.macmillan.org.uk/Cancerinformation/Cancertreatment/Treatmenttypes/Chemotherapy/Individualdrugs/Cisplatin.aspx) | [**C**isplatin](http://www.macmillan.org.uk/Cancerinformation/Cancertreatment/Treatmenttypes/Chemotherapy/Individualdrugs/Cisplatin.aspx) | **O**xaliplatin | **O**xaliplatin |
| [**F**luorouracil](http://www.macmillan.org.uk/Cancerinformation/Cancertreatment/Treatmenttypes/Chemotherapy/Individualdrugs/Fluorouracil.aspx) (also known as 5FU) | capecitabine, which is also known as **X**eloda | [**F**luorouracil](http://www.macmillan.org.uk/Cancerinformation/Cancertreatment/Treatmenttypes/Chemotherapy/Individualdrugs/Fluorouracil.aspx) (also known as 5FU) | capecitabine, which is also known as **X**eloda |

**How is treatment given?**

Chemotherapy is usually given as a course of several sessions (or cycles) of treatment over a few months. Treatment A consists of 3 cycles of chemotherapy pre- and post-surgery with each cycle lasting 21 days (3 weeks). As part of each cycle of ECF/ECX or EOF/EOX you would receive

- **Epirubicin,** which is administered intravenously (through a needle/drip directly into the vein), ***on day 1 of each cycle*** followed by;
- **Cisplatin,** which is administered intravenously (through a needle/drip directly into the vein), ***on day 1 of each cycle***

**or**

- **Oxaliplatin,** which is administered intravenously (through a needle/drip directly into the vein), o*n day 1 of each cycle*, followed by;
- **Fluorouracil (5FU),** which is administered through a tube (details in the 5FU section below) as a continuous infusion ***over the full 21 days of each cycle***.

**or**

- **Capecitabine (Xeloda),** which is given as a tablet, ***twice daily on all 21 days of each cycle****.*

Usually 3 cycles of chemotherapy are given over a period of about 3 months. Treatment can usually be given to you as a day patient on day 1 of each cycle, though a short stay in hospital may be required. The treatment is then continued at home where either Fluorouracil or Capecitabine (Xeloda)|iisis is administered continuously. Further information on how 5FU and capecitabine are given is below.

**Fluorouracil (5FU):**

Most people who receive 5FU will have their chemotherapy given through a thin plastic tube inserted in a vein in the crook of their arm called a PICC (peripherally inserted central catheter) line. |The PICC is either stitched or taped firmly to your arm, and can be kept in the vein for many months.

5FU is delivered to the PICC line through a small portable pump. The pump is attached to the PICC line and is used to give a controlled amount of the drug into your bloodstream over a set period of time while you are at home. You will need to have the drug in the pump, or the entire pump itself, replaced when it empties. This is done in the hospital, usually every week. Before you go you will be given instructions on how to look after the pump. Your nurse should explain how to care for it and what to do if you have any problem with it.

**Capecitabine:**

Capecitabine tablets are taken twice daily for 21 days for each cycle, with no break. It's important to follow the instructions carefully and take the tablets as directed by your doctor, chemotherapy nurse or pharmacist.

**Schedule for Treatment A:**

| WEEK 1 | Cycle 1 of chemotherapy |
| --- | --- |
| 2 |  |
| 3 |  |
| 4 | Cycle 2 of chemotherapy |
| 5 |  |
| 6 |  |
| 7 | Cycle 3 of chemotherapy |
| 8 |  |
| 9 |  |
| 13-15 | Pre-surgery Schedule:  Endoscopy test (OGD)  CT or PET/CT Scan  Standard blood tests  Standard heart and lung tests  Meet study doctor for a decision regarding surgery |

**What will my treatment involve if I am allocated to receive treatment B?**

If you have been randomly assigned to treatment B of the study, you will receive 5 cycles of chemotherapy ***and*** four and a half weeks of radiotherapy ***before*** your proposed surgery.

Radiotherapy treats cancer by using high-energy rays to destroy the cancer cells while doing as little harm as possible to normal cells. This is done by a radiotherapy machine which is programmed for each patient to specifically target cells in the area requiring treatment. The radiotherapy machine itself does not touch you and you don’t feel any pain during the treatment, but you may get some discomfort or pain later on from the side effects. Over the four and a half weeks you will receive 23 radiotherapy treatments.

Chemotherapy given alongside [radiotherapy](http://www.macmillan.org.uk/Cancerinformation/Cancertypes/Gulletoesophagus/Treatingoesophagealcancer/Radiotherapy.aspx) is known as **chemoradiation**. Giving chemotherapy at the same time as radiotherapy may improve the effectiveness of radiotherapy. However, giving both chemotherapy and radiotherapy treatments at the same time may also increase the side effects.

The next section of this patient information leaflet describes the chemotherapy drugs used, the type of radiotherapy used, how they are given and some of the possible side effects. You will receive an information booklet on chemotherapy from your study nurse specialist. You will also be given an information booklet on radiotherapy from your study doctor or nurse specialist. These booklets are given as part of standard care.

**What drugs are used as part of treatment B?**

The chemotherapy portion of treatment 2 consists of two drugs: **Paclitaxel** and **Carboplatin**

Paclitaxel is also called Taxol, so this chemotherapy regimen is sometimes referred to as TaxolCarbo or CarboTaxol. There are no drugs involved in radiotherapy treatment.

**How is chemoradiation treatment given?**

***Chemotherapy***

Prior to each cycle of chemotherapy you will need to have a blood test– either on the same day, or a few days beforehand. You will also be seen by a study doctor, specialist nurse or pharmacist. If the results of your blood test are normal, the hospital pharmacy will prepare your chemotherapy drugs. All of this may take a few hours.

Your chemotherapy treatment may be given to you as a day patient or during a short stay in hospital. When you begin your treatment the nurse will put a thin, flexible tube (cannula) into a vein in your hand or arm. Some people have their chemotherapy given through a thin plastic tube, which is passed through a line in your arm (PICC line, as mentioned previously) |.

Prior to the administration of the chemotherapy agents you will be given anti-sickness drugs (anti-emetics). These are usually given by injection through the cannula, or PICC line, which are connected to a drip (infusion). Some anti-sickness medicines may be given as tablets. You will also be given medicines to prevent an allergic reaction that can occur in some people who receive paclitaxel. This anti-allergy medicine is often given by injection, but part of it is sometimes given as tablets that you take at home before coming to the hospital. It is very important to take the tablets, and you must tell your doctor or nurse if for any reason you haven't taken them.

The chemotherapy drugs are then given one after the other:

- Taxol (a colourless fluid) is given as a drip (infusion) over about one hour.
- Carboplatin (a colourless fluid) is also given as an infusion, over about one hour.

The whole treatment takes approximately 4–5 hours.

If you are having your treatment as a day patient you can then go home, and the cannula will be removed before you go. If you have a PICC line it will usually stay in place, ready for the next cycle of your chemotherapy. You will be shown how to look after the PICC line.

You will be given a supply of anti-sickness tablets to take with you. You should take these regularly if you have been told to do so, even if you are not feeling sick. This is because some medicines are much more effective at preventing sickness than they are stopping it once it has started.

5 cycles of chemotherapy treatment will be given. Each cycle will last one week (7 days). On the first day of each cycle you will be given Taxol and carboplatin. After this you will have a break of 6 days and no further chemotherapy drugs will be given for the remainder of the cycle.

The 1st cycle of chemotherapy will begin on day 1 and the next cycles will begin on days 8, 15, 22 and 29. This makes up the course of chemotherapy treatment.

***Radiotherapy***

Prior to starting your radiotherapy treatment, you will attend the hospital for a CT. This is standard for all patients who will receive radiotherapy for cancer of the oesophagus. Your radiotherapy is planned from this CT. At this visit you will be asked to lie on your back under the CT scan machine. You will have a number of x-rays taken at this time. The radiation therapist (the person who gives you your treatment) will make small permanent marks on your chest area. This is to mark out the area that needs to receive radiotherapy. After this visit your consultant radiation oncologist and the physics team will design and plan your radiotherapy treatment. This process takes approximately two weeks.

As with chemotherapy, radiotherapy involves a course of treatment sessions. A specific amount of radiation is delivered over the full course of treatment; it is divided into a number of smaller doses called fractions. You have the fractions as a series of treatment sessions which make up your radiotherapy course. This allows healthy cells to recover between treatments. Patients receiving radiotherapy as part of treatment B in this study will have 23 fractions (or sessions) totalling 41.4 Gy of radiation. This total dose of radiation is equal to 1.8 Gy per fraction (session) for 4 and a half weeks. The treatment is given in the hospital radiotherapy department as short daily sessions from Monday to Friday. There is no radiotherapy treatment given at the weekend.

You will receive radiotherapy treatment at the same time as the chemotherapy treatment. The 5 weekly cycles of chemotherapy will run concurrently alongside the four and a half weeks of radiotherapy. The radiotherapy sessions in a treatment week are usually given over consecutive days in a row, although days may be interrupted at the discretion of your radiation therapist or due to planned treatment days running over a weekend.

The 1^st^ cycle of radiotherapy will also begin on day 1 of chemotherapy cycle 1. The duration of your radiotherapy is at the discretion of your radiologist but usually is given for four and a half weeks as follows: days 1-5, days 8-12, days 15-19, days 22-26 and days 29-31 inclusive.

The treatment schedule in the next section provides a clear visual representation of how the chemotherapy and radiotherapy portions of treatment B are given.

##### **Schedule for Treatment B:**

| WEEK 1 | Cycle 1 Chemo day 1 | Radiation days 1-5 |
| --- | --- | --- |
| 2 | Cycle 2 Chemo day 8 | Radiation days 8-12 |
| 3 | Cycle 3 Chemo day 15 | Radiation days 15-19 |
| 4 | Cycle 4 Chemo day 22 | Radiation days 22-26 |
| 5 | Cycle 5 Chemo day 29 | Radiation days 29-31 |
| 9-11 | Pre-surgery Schedule:  Endoscopy test (OGD)  CT or PET/CT Scan,  Standard blood tests  Standard heart and lung tests  Meet study doctor for a decision regarding surgery |  |

What are the possible risks or discomforts involved in study treatment?

***Chemotherapy side effects***

Each person's reaction to chemotherapy is different. Some people have very few side effects, while others may experience more. The side effects described below will not affect everyone who is having this treatment. We have outlined the most common side effects and some of the less common ones, so that you can be aware of them if they occur. We have not included those that are rare and therefore unlikely to affect you. If you notice any effects that you think may be due to the drug, but which are not listed here, please discuss them with your study doctor, chemotherapy nurse or pharmacist.

Very common side effects, occurring in 1 in 10 patients (10%) or more, include:

- Lowered resistance to infection**;** while you are having chemotherapy the production of white blood cells by the bone marrow is reduced, making you more prone to [infection](http://www.macmillan.org.uk/Cancerinformation/Livingwithandaftercancer/Symptomssideeffects/Othersymptomssideeffects/Avoidinginfection.aspx)|. During the period when you aren't having chemotherapy (or while you are only having 5FU through the pump) this will begin to recover. It will usually have returned to normal before your next cycle of chemotherapy. You may have headaches, aching muscles, a cough, a sore throat, pain passing urine, or you may feel cold and shivery. **Contact your treatment centre straight away if you have any of these effects or if your temperature goes above 38°C.**
- Anaemia (low number of red blood cells); while having treatment with ECF/ECX you may become anaemic. This may make you feel [tired](http://www.macmillan.org.uk/Cancerinformation/Livingwithandaftercancer/Symptomssideeffects/Fatigue/Fatigue.aspx)| and [breathless](http://www.macmillan.org.uk/Cancerinformation/Livingwithandaftercancer/Symptomssideeffects/Othersymptomssideeffects/Breathlessness.aspx)|.
- Bruising or bleeding; ECF can reduce the production of platelets (which help the blood to clot and stop bleeding). You may have unexplained bruising or bleeding, such as nosebleeds, blood spots or rashes on the skin, and bleeding gums.

**The side effects above can be life threatening, particularly infections. If you think you are experiencing any of these side effects, you should contact your study doctor or nurse immediately.**

- Tiredness and feeling weak; many people feel extremely [fatigued (tired)](http://www.macmillan.org.uk/Cancerinformation/Livingwithandaftercancer/Symptomssideeffects/Fatigue/Fatigue.aspx)| during chemotherapy, particularly towards the end of treatment.
- Discoloured urine; your urine may become a pink-red colour after epirubicin treatment. This is due to the colour of the drug and may last for up to a day after the treatment
- Feeling sick (nausea) and being sick (vomiting) is most likely in the few days after treatment.
- Diarrhoea; if you have [diarrhoea](http://www.macmillan.org.uk/Resourcessupport/Eatingwell/Eatingproblems/Diarrhoea)| it can usually be easily controlled with medicine. Let your study doctor know if it is severe (more than 4-6 times a day). It is important to drink plenty of fluids if you have diarrhoea.
- Hair loss; this usually starts 3–4 weeks after the first course of treatment. Hair usually falls out completely. You may also have thinning and loss of eyelashes, eyebrows and other body hair. [Hair loss](http://www.macmillan.org.uk/Cancerinformation/Livingwithandaftercancer/Symptomssideeffects/Hairloss/Hairloss.aspx)| is temporary and your hair will start to grow again once the treatment has finished. Your nurse can give you advice about coping with hair loss.
- Skin colouration changes; during chemotherapy treatment; your skin may darken in colour, this will slowly return to normal a few months after the treatment has finished.
- Skin rashes and itchiness; your skin may become itchy and dry.
- Soreness and redness on the palms of your hands and soles of your feet (known as hand-foot syndrome or palmar-plantar syndrome); this is temporary and improves when the treatment is finished.
- Loss of fertility; your ability to become pregnant or father a child may be affected by this treatment. It's important to discuss [fertility](http://www.macmillan.org.uk/Cancerinformation/Livingwithandaftercancer/Symptomssideeffects/Fertility/Fertility.aspx)| with your study doctor before starting treatment.
- Loss of appetite; the dietician can give you tips on boosting your appetite, coping with eating difficulties and maintaining weight.
- Loss of periods in women (amenorrhoea); Due to the effect of chemotherapy on the ovaries you may find that your periods become irregular and may eventually stop. In younger women this may be temporary, but if you are closer to your menopause it may be permanent. This will result in menopausal symptoms such as hot flushes, sweats and vaginal dryness.
- Harmful effects on a developing foetus; it is not advisable to become pregnant or to father a child while receiving this treatment. It is important to use an effective and medically recommended barrier method of contraception while taking these drugs, and for at least 6 months afterwards. Your nurse or doctor will discuss appropriate methods or a combination of methods for you if required.
- Sensitivity of the skin to sunlight; during treatment and for several months afterwards, you will be more sensitive to the sun, and your skin may burn more easily than normal. You can still go out in the sun, but always wear a high protection factor sun cream, protective clothing and a hat.
- **Aching joints and muscles;** you may have these symptoms a few days after treatment (due to the Taxol). This does not usually last long and your study doctor can prescribe mild painkillers to help.
- **Allergic reaction;** some people can have an allergic reaction to the chemotherapy during the infusions. Signs of an allergic reaction include skin rashes and itching, a high temperature, shivering, redness of the face, a feeling of dizziness, headache, breathlessness, anxiety and a desire to pass urine. You will be monitored for any signs of an allergic reaction during the treatment. Tell your study doctor or nurse if you have any of these signs. To help prevent the chance of an allergic reaction you will be given steroid|  tablets (usually dexamethasone) to take at home a number of hours before the paclitaxel. It's important to take them as directed and to tell your study doctor or nurse if you forget. Instead of tablets the dexamethasone can sometimes be given by injection into a vein (intravenously) 30-60 minutes before the paclitaxel. As well as the steroid, 30-60 minutes before the paclitaxel you will be given intravenous injections of an antihistamine, which helps to prevent an allergic reaction, and an antacid, which helps to stop the steroid damaging your stomach.
- Mild allergic reactions occur in more than 3 out of 10 people (34%) this usually shows as a rash or a red face
- Low blood pressure during treatment; your blood pressure will be checked regularly. Tell your study doctor or nurse if you feel faint or dizzy.
- Alcohol intoxication effects; you may feel a bit ‘tipsy’ because the paclitaxel drip contains alcohol to help dissolve the drug – it is the equivalent of a large glass of wine or a pint of beer
- **Effect on your kidneys**; this is unlikely to cause any symptoms. The function of your kidneys will be checked by a blood test before each cycle of chemotherapy treatment. Sometimes a 24-hour urine test is also carried out.

Occasional side effects, occurring in between 1 in 100 (1%) to 1 in 10 (10%) patients, include:

- Inflammation around the drip site; caused by leakage of drug into the tissue around the vein, can damage the tissue in that area. This is unlikely to happen if the chemotherapy is given through a central line or PICC line.
- Changes in nails; your nails may become darker and white lines may appear on them. These changes usually grow out over a few months once the treatment has finished.
- Sore eyes
- Watery eyes
- ‘Gritty’ feeling eyes and blurred vision
- Numbness or tingling in hands or feet; this is due to drug effects on nerves and is known as [peripheral neuropathy](http://www.macmillan.org.uk/Cancerinformation/Livingwithandaftercancer/Symptomssideeffects/Othersymptomssideeffects/Peripheralneuropathy.aspx)|. This problem usually improves slowly a few months after treatment is over.
- Taste changes; you may notice that your food tastes different, notice a loss of taste, or experience a metallic taste in your mouth. Normal taste will usually come back after the treatment finishes.
- Damage to heart muscle; higher doses of epirubicin may cause changes in the muscle of the heart which can affect how your heart works. It's unusual for the heart to be affected if you have standard doses. Tests to see how well your heart is working may sometimes be carried out before the drug is given.
- Ringing in the ears (tinnitus) usually improves when treatment ends.
- Changes in hearing; you may lose the ability to hear some high-pitched sounds. Hearing loss can be more severe with higher doses and longer courses of treatment. Very occasionally your sense of balance may be affected. Any hearing loss and balance changes if they occur are likely to be permanent.
- Abdominal pain and constipation; It may help to drink plenty of fluids, eat a high fibre diet and take gentle exercise. Sometimes you may need to take medicines to stimulate your bowel. These can be prescribed by your doctor.
- Sore eyes
- Watery eyes
- Blurred vision
- Increased production of tears; this is a temporary side effect, although the tears can sometimes irritate the front of the eye.
- Difficulty swallowing or breathing can be triggered by cold air in the first 5 days after you have oxaliplatin but usually clears up on its own – tell your doctor or nurse if you have this side effect and avoid cold drinks or ice cubes for the first few days
- Allergic reaction; signs of an allergic reaction include skin rashes and itching, a high temperature, shivering, redness or darkening of the face, dizziness, headaches, breathlessness, anxiety, and a need to pass urine. You will be monitored for any signs of an allergic reaction during the treatment.
- Slowing of the heart rate (bradycardia); paclitaxel can sometimes cause temporary changes in heart rate. This usually does not cause any harm.
- Abdominal pain; this may start a few days after finishing the chemotherapy and may last for a few days. Your study doctor can prescribe regular painkillers if this happens
- Your liver may be temporarily affected; paclitaxel may cause changes in the way that your liver works, though your liver will return to normal when the treatment is finished. This is very unlikely to cause you any harm, but your study doctor will monitor this carefully. Samples of your blood will be taken from time to time to check that your liver is working properly.

***Radiotherapy side effects***

Radiotherapy can cause general side effects|. It is not unusual to feel worse before things start to feel better. Some of the side effects occur immediately, some after treatment is finished and others can occur months or years later. Your study doctor will be able to advise you on what to expect. Immediate side effects include:

- Feeling sick/nausea: You may feel sick or nauseous during your treatment. Feeling sick can usually be treated by anti-sickness drugs, which your study doctor can prescribe.
- Sore throat and difficulty swallowing: Radiotherapy to the oesophagus can cause inflammation of the throat and oesophagus, as the cells that line these areas are very sensitive to treatment. The radiotherapy may make your throat or mouth very sore towards the end of your course of treatment, and you [may not be able to swallow properly](http://www.macmillan.org.uk/Cancerinformation/Cancertypes/Gulletoesophagus/Livingwithgulletcancer/Difficultyinswallowing.aspx) for a while. It may feel like you have a lump in your throat. It is very important that you inform your study doctor or nurse if you experience any problems swallowing.
- Dry mouth: If the radiotherapy is given to the upper end of the oesophagus, it may reduce the amount of saliva your salivary glands produce. This effect may be temporary, but occasionally it can be permanent. This may make your mouth dry, which can make eating difficult. It is advised to drink fluids at regular intervals to keep your mouth moist.
- Reduced appetite and weight loss: Sometimes if it is the part of your oesophagus near your mouth that requires treatment, it can affect the taste buds in your mouth. You may experience a loss of taste or a metallic taste in your mouth. If this happens you may lose interest in food and not eat as much as you did before treatment began. If you don't feel like eating, you can replace meals with nutritious, high-calorie drinks. Your appetite should improve once treatment ends.
- Hair loss: When radiotherapy is used to treat the oesophagus, men may find that some of the hair on their chest may fall out. The hair loss is usually temporary but any future hair growth may be thinner than it was before.
- Fatigue and Tiredness: As radiotherapy can make you [tired](http://www.macmillan.org.uk/Resourcessupport/Symptomssideeffects/Fatigue), you should try to get as much rest as you can.
- Skin Changes: During radiotherapy the skin in the affected area may become red and sore. It may look like sunburn. Your study doctor may recommend a topical cream to help.
- Voice Changes: Occasionally, radiotherapy to the oesophagus may cause the sound of your voice to change. Your voice may become quieter or sound hoarse. It may be sore for you to talk. This is usually temporary and the quality of your voice should start to improve about 2-3 weeks after treatment.

All these side effects should disappear gradually once your course of treatment is over, but it is important to let your study doctor know if they continue. Long term side effects include:

- Skin Changes: The most common long-term side effect is a change to the skin in the treated area. It may become coarser in texture or look darker in colour.
- Damage to heart: The heart and major blood vessels can be damaged by radiotherapy. The risk is very small as we do our best to very carefully minimise the radiation dose to these structures.
- Lung Damage: Unavoidably some normal lung tissue may be damaged. This is not usually a problem, but in the long term you may find that you are a little more breathless than you used to be. Let your study doctor know if this is a problem for you.
- Inflammation of lung tissue (radiation pneumonitis); is a rare but potentially serious side-effect of chest radiotherapy. Symptoms are cough and increasing shortness of breath, usually about six weeks after Radiotherapy. Please inform your study doctor immediately if you experience these symptoms.
- Narrowing of the oesophagus Radiotherapy may cause your oesophagus to narrow in the long term, causing a “stricture”. You may find food may “stick” in your throat and you may need a procedure to stretch it. Let your study doctor know if you have any increased problems with swallowing food.

***Side effects of combined chemotherapy and radiotherapy (chemoradiation)***

Having radiotherapy and chemotherapy together can be quite intensive. Chemotherapy in combination with radiotherapy is likely to cause greater side effects than chemotherapy or radiotherapy alone. Radiotherapy, especially combined with chemotherapy can lead to oesophagitis, which is a term for any inflammation, irritation, or swelling of the oesophagus.

**What are the possible benefits from taking part in this study?**

It is well established that often the best cancer outcomes are obtained within well-designed and conducted clinical research studies; these are subjected to rigorous quality assurance. Other than a commitment to excellent multidisciplinary care, there is no way of knowing if you will derive any benefit from being in the study. The aim of the study is to determine if either treatment shows an improvement in outcomes for this type of cancer, or whether the treatments are equal.

Whilst you are taking part in this study you will get more intensive medical supervision throughout your treatment and during the follow up period. It is hoped that the treatments you receive during this study will cure your cancer, but this cannot be guaranteed and your participation in this study may be of no personal benefit to you. The information we learn from this study will hopefully benefit patients suffering from cancer of the oesophagus in the future**.**

**What if new information becomes available?**

Sometimes during the course of a research study, new information becomes available about the treatments being studied. If this happens, we will tell you or your legally acceptable representative about it and discuss with you whether you want to continue in the study. If you decide to withdraw from the study your study doctor will make arrangements for your further care.

Also, on receiving new information your study doctor might consider it to be in your best interests to withdraw you from the study. If this happens, he/she will explain the reasons and arrange for your care to continue.

**Can I stop being in the study?**

Yes, you can decide to stop at any time. Tell the study doctor if you are thinking about stopping or decide to stop. He or she will tell you how to stop safely.

It is important to tell the study doctor if you are thinking about stopping so any risks from the study drugs can be evaluated by your study doctor. Another reason to tell your study doctor that you are thinking about stopping is to discuss what follow-up care and testing could be most helpful for you.

You can choose to withdraw one of two ways. In the first instance, you can stop your study treatment, but still allow the study doctor to follow your care. In the second instance, you can stop your study treatment and not have any further contact with the study staff.

**Can anyone else stop me from being in the study?**

The study doctor may stop you from taking part in this study at any time if;

- it is in the best interest for your health,
- You experience severe or life-threatening side effects. The study treatment will be stopped if serious side effects develop, and appropriate medical care will be provided.
- you do not follow your responsibilities for taking part in the study,
- it is discovered at a later time that you do not meet the study participation requirements
- you become pregnant
- you need treatment not allowed in the study
- your disease becomes worse. If your disease becomes worse during treatment, you will be told, and the treatment will be stopped. Other medical care will be discussed with you.
- the study is stopped by the sponsor or regulatory authority <name of the applicable Regulatory Authority>.
- the sponsor stops enrolling new patients for any reason and you are in the screening phase and not yet assigned to a treatment group.
- administrative reasons require your withdrawal

**What happens if I am injured because I took part in this study?**

It is important to note that nothing said in this consent form alters your legal rights to seek to recover damages should you suffer an injury as a result of participation in this study. Every reasonable precaution will be taken to ensure your safety during the course of the study.

Participation in this study is covered by an approved policy of insurance in the name of ICORG. In addition, the medical practitioners involved in this study have current medical malpractice insurance and coverage under the current Clinical Indemnity Scheme. The Sponsor will comply with the Irish Pharmaceutical Healthcare Industry (IPHA) guidelines and Irish Law (statutory and otherwise) in the unlikely event of your becoming ill or injured as a result of participation in this clinical study. The amount of any compensation paid may, however, be reduced if you have not complied with the instructions given for the study.

It is important that you tell your study doctor, if you feel that you have been injured because of taking part in this study. You can tell the study doctor in person or call him or her at ___________________.

**Will my taking part in this study be kept confidential?**

Every effort will be made to ensure that the personal information in your medical record will be kept private. If information from this study is published or presented at scientific meetings, your name and other personal information will not be used. The information collected, as part of this study will be shared with other researchers and doctors. If you consent to take part in the study any of your medical records may be inspected by the company organising the research for purposes of analysing the results. They may also be looked at by people from regulatory authorities to check that the study is being carried out correctly. However strict confidentiality will be maintained at all times. Organisations that may look at and/or copy your medical records for research, for quality assurance, and data analysis include:

- ICORG
- Regulatory agencies such as the Health Products Regulatory Authority (formerly the Irish Medicines Board) and/or their designated representatives or other regulatory agencies
- Department of Health (DoH)
- Research Ethics Committee (SJH/AMNCH Research Ethics Committee)

**What are the costs of taking part in this study?**

You will not be charged for the cost of tests done for the purpose of this study. You will not be paid for your participation in this study. Your travel expenses will not be reimbursed.

**Who has reviewed and approved this study?**

This study has been approved by SJH/AMNCH Research Ethics Committee and the Health Products Regulatory Authority (formerly the Irish Medicines Board).

**Contact for further information**

If you have any questions concerning the procedures of this study, or if any problems arise during the Clinical Research Study, you should contact the following people:

Study Doctor Name: ___________________ Telephone: ___________________

For questions about your rights or if you wish to make a complaint while taking part in this study, call the Clinical Trials Manager, Beaumont Hospital at (01) 809 2373,

The Hospital Complaints Department, St James Hospital at 01-4103361, Director of Nursing Office, University College Hospital, Galway at 091-544465, Risk Management Office, Cork University Hospital at 021-4922822 or Risk Management Office, Mercy Hospital Cork at (021) 4271971.

**INFORMED CONSENT FORM**

**Study Title: Neo-AEGIS:** Randomised trial in adenocarcinoma of the oesophagus and oesophago-gastric junction: A comparison of neo-adjuvant and adjuvant chemotherapy and surgery versus neo-adjuvant chemoradiation and surgery**.**

**Study Doctor Name: __________________ Hospital name: ___________________**

**Please initial box**

1. I confirm that I have been given a copy of the Patient Information Leaflet and Consent form ICORG Version X, dd-mmm-yyyy, Hospital Name Version X: dd-mmm-yyyy. I have read the Patient Information Leaflet and Consent Form or it has been read to me. This information was explained to me and my questions were answered.

2. I understand that my participation is voluntary and that I am free to withdraw at any time without giving any reason and without my medical care or legal rights being affected.

3. I understand that relevant parts of my medical records may be seen by ICORG, The Research Ethics Committee, Regulatory Authorities and all organisations as listed in this Informed Consent Form provided they agree not to disclose my name.

4. I understand that data related to me collected during this study will be processed and analysed as is required by this clinical study and according to the Data Protection Act.

5. I agree to my GP being informed of my involvement in this study

6. I agree to take part in the above study.

______________________ __________________ _____________

Name of Patient (Print) Signature of Patient Date

______________________ __________________ _____________

Name of Witness (Print, Signature of Witness Date

(IF APPLICABLE)

______________________ __________________ _____________

Name of Study Doctor (Print) Signature of Study Doctor Date

______________________ ____________________ ____________

Name of Research Nurse Signature of Research Nurse Date

(Print, IF APPLICABLE)
