# Supplementary material for: ICORG 10-14: NEOadjuvant trial in Adenocarcinoma of the oEsophagus and oesophagoGastric junction International Study (Neo-AEGIS)
Source: BMC Cancer. 2017 Jun 3;17:401. doi: 10.1186/s12885-017-3386-2 (PMC5457631; doi:10.1186/s12885-017-3386-2)
Supplement: Supplementary file 2 — Appendix B: - Description of data: Membership of Trials Steering Committee (DOCX 22 kb) [file 12885_2017_3386_MOESM2_ESM.docx]

**Appendix B: Membership of Trial Steering Committee**

Kathleen Scott, ICORG Clinical Programme Leader

John Reynolds, Chief Investigator, Consultant Surgeon, Ireland

Lene Baeksgaard, PI, Consultant Surgeon, Denmark

Christophe Mariette, PI, Consultant Surgeon, France

Shaun Preston, PI, Consultant Surgeon, UK

Sinead Cuffe, Consultant Medical Oncologist, Ireland

Brian O’Neill, Consultant Radiation Oncologist, Ireland

Tom Crosby, PI, Consultant Surgeon, UK

Ray McDermott, Vice-Clinical lead, ICORG

Imelda Parker, Statistician, ICORG

Michele Cunnane, Data management lead, ICORG

Paulina Lawner, Clinical Project Manager, ICORG

Mary Stapleton, Pharmacovigilance, ICORG

Jane Mellor, Clinical Trials Manager, SCTU UK

Kerry-Ann Lee, Clinical Trials Co-ordinator, SCTU UK

Non-voting member:

Grainne O’Dowd, ICORG Clinical Research Associate
